# A New Mouse Model of Mild Ornithine Transcarbamylase Deficiency (*spf-j*) Displays Cerebral Amino Acid Perturbations at Baseline and upon Systemic Immune Activation

**DOI:** 10.1371/journal.pone.0116594

**Published:** 2015-02-03

**Authors:** Tatyana N. Tarasenko, Odrick R. Rosas, Larry N. Singh, Kara Kristaponis, Hilary Vernon, Peter J. McGuire

**Affiliations:** 1 National Human Genome Research Institute, National Institutes of Health, Bethesda, Maryland, United States of America; 2 Universidad Central de Caribe, Bayamon, Puerto Rico, United States of America; 3 Department of Neurogenetics, Kennedy Krieger Institute, Johns Hopkins University, Baltimore, Maryland, United States of America; 4 Kennedy Krieger Institute, Johns Hopkins University, Baltimore, Maryland, United States of America; National Institute of Agronomic Research, FRANCE

## Abstract

Ornithine transcarbamylase deficiency (OTCD, OMIM# 311250) is an inherited X-linked urea cycle disorder that is characterized by hyperammonemia and orotic aciduria. In this report, we describe a new animal model of OTCD caused by a spontaneous mutation in the mouse *Otc* gene (c.240T>A, p.K80N). This transversion in exon 3 of ornithine transcarbamylase leads to normal levels of mRNA with low levels of mature protein and is homologous to a mutation that has also been described in a single patient affected with late-onset OTCD. With higher residual enzyme activity, *spf-J* were found to have normal plasma ammonia and orotate. Baseline plasma amino acid profiles were consistent with mild OTCD: elevated glutamine, and lower citrulline and arginine. In contrast to WT, *spf-J* displayed baseline elevations in cerebral amino acids with depletion following immune challenge with polyinosinic:polycytidylic acid. Our results indicate that the mild *spf-J* mutation constitutes a new mouse model that is suitable for mechanistic studies of mild OTCD and the exploration of cerebral pathophysiology during acute decompensation that characterizes proximal urea cycle dysfunction in humans.

## Introduction

Ammonia is a normal constituent of all body fluids and is generated from the catabolism of nucleotides and amino acids [1]. This neurotoxin is converted to water-soluble urea by the urea cycle for excretion in the urine. Urea cycle disorders (UCD) are caused by loss of function in any of a group of enzymes responsible for ureagenesis and may be characterized by chronic and acute hyperammonemia (HA)[2]. UCD can be considered as proximal disorders in which ammonia disposal is severely compromised, or as distal disorders, in which ammonia disposal is not as severely impaired and characteristic amino acid metabolites accumulate.

Hyperammonemic coma in UCD can result in severe brain injury. The mechanisms of injury are varied and include changes in neurotransmitters, cerebral edema secondary to elevated glutamine and disruption of mitochondrial energy metabolism [3]. The primary morbidity for UCD patients who survive their hyperammonemic events is neurological. The proportion of UCD patients with significant intellectual deficits has been estimated to range from 50–80% [4,5]. This intellectual disability may be due to hyperammonemic insults in the newborn period or even episodic hyperammonemia [6,7]. In addition to neurologic insults secondary to hyperammonemic events, asymptomatic carriers of the most common UCD, ornithine transcarbamylase deficiency (OTCD), may have deficits in non-verbal learning, fine motor processing, reaction time, visual memory, attention, and executive function [8]. Moreover, asymptomatic OTC carriers can show changes in brain metabolites including elevated glutamic acid and glutamine, and depleted choline and myoinositol [9]. These findings suggest that alterations in brain neurochemistry may be behind the neurocognitive deficits seen in asymptomatic OTC.

Ornithine transcarbamylase deficiency (OTCD, OMIM 311250) is an X-linked disorder and the most common form of inborn error of the urea cycle. Males with OTCD often present in the newborn period with profound hyperammonemia requiring extracorporal removal of ammonia, while females may range from neonatal to late-onset to asymptomatic disease due to X-inactivation status in the liver [10,11]. Milder alleles independent of X-inactivation status have also been reported [12,13]. Biochemically, OTCD is characterized by elevated plasma and urine concentrations of ammonia, glutamine, and orotic acid, with downstream arginine deficiency [10,11]. Treatment of patients affected with OTCD centers on the avoidance of hyperammonemia through protein restriction, reversal of catabolism, L-citrulline therapy, and enhanced nitrogen disposal by alternative pathways [11].

To date, the only gene associated with OTCD is *OTC*, located on chromosome Xp21.1. This gene encodes ornithine transcarbamylase, a mitochondrial enzyme that catalyzes the second step in the urea cycle, in which carbamoyl phosphate and ornithine are condensed to form citrulline. Many genetic alterations have been reported at this locus (http://ureacycle.cnmcresearch.org/otc/page6/page7/page7.html) including deletions, duplications, nonsense, and missense mutations. Genotype/phenotype correlations have been described, with some variation [14]. In general, mutations that abolish enzyme function are associated with a relatively uniform clinical and biochemical presentation.

Animal models of OTCD that have been characterized and are readily available include the *spf* mouse and the *spf-ash* mouse. These models are currently maintained on a mixed background, *B6EiC3Sn*, which may limit certain issues of experimental design. Herein, we describe a new spontaneous hypomorphic missense mutation in the C57BL/6J-*Otc^spf-J^*/J (*spf-J*) that produces a new model of mild OTCD. *Spf-J* mice display normal plasma ammonia and plasma orotic acid at baseline and milder amino acid perturbations when compared to their predecessors. Despite this mild plasma biochemical phenotype, cerebral amino acid concentrations were elevated at baseline and, unlike WT, were depleted during a systemic immune response suggesting altered cerebral amino acid metabolism or transport. Overall, this mouse model is not only useful for further characterization of functional domains of the OTC enzyme, but is also useful for investigating the pathophysiology of cerebral amino acid metabolism in UCD.

## Methods

### Animal care and experimental interventions

The experiments outlined were performed on 8-week-old C57BL/6J-*Otc^Spf-J^*/J (*spf-J*) and wildtype (WT) littermates (The Jackson Laboratory, Bar Harbor, ME). Mice were housed in a pathogen-free facility, caged individually and had access to a 22% protein pellet based feed, (Bio-Serv, Frenchtown, NJ) as well as autoclaved reverse osmosis water. Mice were housed 5 animals/cage in a temperature (22 + 2°C) and humidity (30–70%) controlled environment with a 12-hour light cycle. Veterinary and research staff were responsible for the monitoring of mice. For the high protein diet challenge, mice were randomized to groups containing maintained 22% or 70% protein (Harlan, Madison, WI) for 2 weeks before euthanasia. An immune challenge was performed by intraperitoneal injection of polyinosinic:polycytidylic acid (100 μg) once a day for 3 days. Post immune challenge, the animals were euthanized by isoflurane anaesthesia followed by cervical dislocation and tissues were harvested and stored at −80°C until analysis. Retro-orbital blood collection was performed following treatment with optic tetracaine hydrochloride (Bausch and Lomb, Rochester, NY). All procedures were approved by the Animal Care and Use Committee of the National Human Genome Research Institute, an Association of Assessment and Accreditation of Laboratory Animal Care (AAALAC) International accredited institution.

### Morbidity study

*Spf-j* and WT animals were monitored two or more times daily by veterinary personnel and/or researchers responsible for their care. The *spf-j* animals described in the morbidity study were found dead in their cages. During their care, they did not display any clinical criteria requiring euthanasia. Animals surviving the study more than 200 days were used for other experiments not related to this paper. The method of euthanasia for all studies was isoflurane anaesthesia followed by cervical dislocation.

### OTC mutation determination and genotyping

The isolation of genomic DNA from mouse tails was performed using DNeasy Blood and Tissue Kit according to the manufacturers instructions (Qiagen, Valenica, CA). Primers encompassing the 10 exons of the OTC gene were designed (sequences available upon request). PCR was performed with BioRad iProof™ High-fidelity DNA Polymerase (BioRad, Hercules, CA) and resulting products were cut from the agarose gel and inserted into pCR™4-TOPO TA vector (Life Technologies, Grand Island, NY) for sequencing. For genotyping, amplification was performed in 20μl with MyTag PCR mix (Bioline, Taunton, MA) for 35 cycles 1 min denaturation at 95 C, 45s annealing at 59 C and 1 min polymerization on 72 c. Oligonucleotide primers used to amplify Exon 3 were F: 5′-GCTTGATGTTGGATAGTGTACCTTGC-3′ and R: 5′-AAGCCTTCCAAGTCTCCATCCTCT-3′. The A to T transversion identified by sequencing in exon 3 was detected using a restriction digest. After DNA amplification, 10μl of PCR product was digested with EcoRI restriction enzyme in a total volume of 20 μl for 1 hour at 37C. Digests were resolve d on 1.2% agarose gel. WT mice were identified by a 207 bp product while *spf-J* have two fragments 128 and 79bp.

### Ammonia, orotate and amino acids

For blood collection, retro-orbital bleeds were performed with collection in lithium heparin microtainers (BD Diagnostics, Franklin Lake, NJ). Plasma ammonia was determined using a VetTest 8008 Chemistry analyzer (Idexx Laboratories, Westbrook, ME). Plasma orotate was measured using liquid chromatography mass spectrometry according to a previously published method [15]. For amino acid analysis of plasma and tissues, ion exchange chromatography with ninhydrin detection was performed using a Biochrom 30 Amino Acid Analyzer (Biochrom, Cambridge, UK). For tissues, amino acid concentrations were expressed as μmol / 100 gram of tissue. For tissue analysis, citrulline was removed due to tissue interference with detection.

### OTC enzyme activity

Ornithine transcarbamylase enzyme activity was measured by a colorimetric assay which detects the formation of L-citrulline [16]. Liver tissue was homogenized in mitochondrial lysis buffer: 0.5% Triton 100-X, 10 mM HEPES sodium salt, 0.5 mM DTT, 2 mM EDTA, and 1% protease inhibitor cocktail (Thermo Scientific, Rockford, IL) pH 7.4. Assays were performed in 50 mM Tris acetate, 2 mM EDTA, pH 8.3 at 25°C and normalized to cellular protein. Results were expressed as μmol citrulline/g protein/hour.

### qRT-PCR for mRNA

RNA was extracted from homogenized liver tissue or cell pellets using RNeasy Minikit (Qiagen, Valencia, CA) according to the manufacturer’s instructions. One μg of RNA was reverse transcribed to cDNA using a modified MMLV-reverse transcriptase with RNase H+ activity (iScript, Bio-Rad, Hercules, CA) according to the manufacturer’s instructions. Real-time quantitative PCR reactions were carried using TaqMan systems (Applied Biosciences, Carlsbad, CA). Reactions were cycled and quantitated with an ABI 7500 Fast Real Time PCR System (Applied Biosystems, Foster City, CA).

### Immunoblotting

For western blot analysis, approximately 30 μg of protein was loaded on 4–20% Tris-glycine polyacrylamide gels. The gels were run at 150 V for approximately 1.5 h. The gels were transferred to polyvinylidene difluoride membrane using the iBlot Dry Blotting System (Life Technologies, Grand Island, NY). The membranes were incubated overnight at 4°C in blocking buffer (LiCor, Lincoln, NE). The membranes were probed with the following primary antibodies according to the manufacturers suggested dilutions: OTC (Novus Biologicals, Littleton, CO) and β-actin (Sigma-Aldrich, St. Louis, MO). The membranes were washed three times for 10 min each with TBS containing 0.1% Tween 20, followed by incubation with appropriate secondary antibodies. Image analyses were performed using an Odyssey Imager (LiCor, Lincoln, NE).

### Statistical analyses

Summary statistics were calculated for all data. Two-sided student’s t-test and survival analysis was used when appropriate. P-values less than 0.05 were considered to indicate statistical significance. Baseline statistical analyses were performed using Prism 5 (GraphPad Software, Ja Jolla, CA) software package. To quantify the difference in poly I:C and baseline amounts of each amino acid, we used a t-statistic for the difference between poly I:C and baseline, and plotted these t-statistics. T-statistics were computed R v3.0.2 (R Core Team, R: A Language and Environment for Statistical Computing, 2013, www.R-project.org), and graphs were plotted using ggplot2 (gglot2.org). To compute the p-value of the significance in differences of change in *spf-j* versus WT, we used a form of permutation test[17] as follows. For each amino acid, let D_spf-j_ represent the mean change in amino acids from baseline to *spf-j*. Therefore, D_spf-j_ is defined as the difference between mean amount of *spf-j* polyI:C amino acid and the mean amount of spf-j amino acid. Likewise, let D_wt_ be the difference between mean amount of WT polyI:C amino acid and the mean amount of WT baseline amino acid. Then the difference in change of amino acid between spf-j and WT can be measured as D = D_spf-j_ * let D_wt_ with the greatest difference in change occuring when D is most negative. D therefore represents our statistic for difference in change from baseline to polyIC. Random permuted values of D_perm_ are computed by permuting the labels of *spf-j*, WT, baseline and polyI:C for each value of amino acid, and re-calculating D. The p-value is computed as the mean number of D_perm_ less than the original, unpermuted D. We performed 100,000 permutations for each p-value computation.

### Ethics statement

All experiments described in this paper were approved by the Animal Care and Use Committee at the National Human Genome Research Institute.

## Results

### Identification of the missense mutation in the *spf-J*

**Figure 1:**
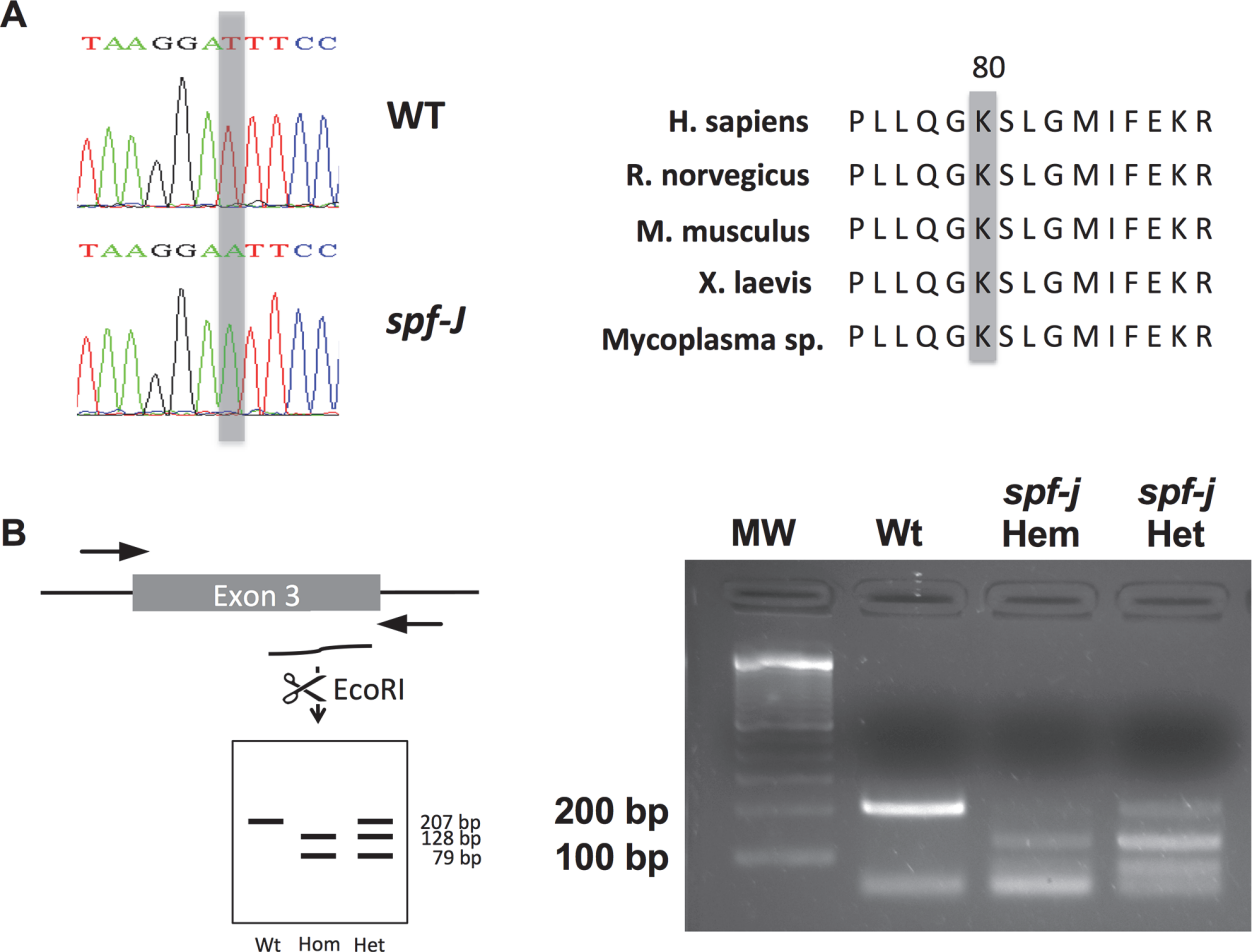
Identification of the molecular lesion in *spf-J*. A) PCR amplification of gDNA from *spf-J* revealed a transversion mutation in exon 3 leading to an asparagine replacement of a conserved lysine residue at position 80 (K80N). B) Genotyping assay of female *spf-J* with PCR amplification followed by digestion with EcoRI. Hem – hemizygote, Het – heterozygote.

OTC was considered the likely candidate for the *spf-J* phenotype based on the results of a non-complementation test with *spf* performed at the Jackson Laboratory [18]. To confirm the hypothesis that the *spf-J* mutant allele involved the OTC gene, OTC-specific primers were designed and used to amplify exons 1–10 of mutant and WT gDNA. PCR products amplified from both mutant and WT mice were of the expected size (data not shown). However, the gDNA sequence for *spf-J* contained a c.240T>A transversion in exon 3 (Fig. 1A), leading to a lysine to asparagine substitution (K80N). An evolutionary comparison of OTC amino acid sequences showed that the K80 residue is located outside the carbamoyl phosphate and ornithine binding regions [13] and is conserved across multiple species (http://blast.ncbi.nlm.nih.gov/Blast.cgi, Fig. 1A). Interestingly, a single patient with late-onset OTC deficiency in adolescence displaying the same mutation as *spf-j* has been described [19].

With the identification of the mutation in exon 3, we next sought to develop a genotyping assay for rapid identification of progeny (Fig. 1B). Scanning exon 3, we identified an allele specific EcoRI site. Fragments amplified by PCR of genomic DNA were digested with EcoRI to yield fragments of 207, 128 and 79 base pairs (bp), allowing us to easily identify WT (207, 79 bp), female heterozygotes (207, 128, 79 bp) and male hemizygotes (128, 79 bp). This genotyping assay was then used for subsequent identification of all male experimental animals.

### Smaller *spf-J* regain weight and display comparable survival to unaffected controls

**Figure 2:**
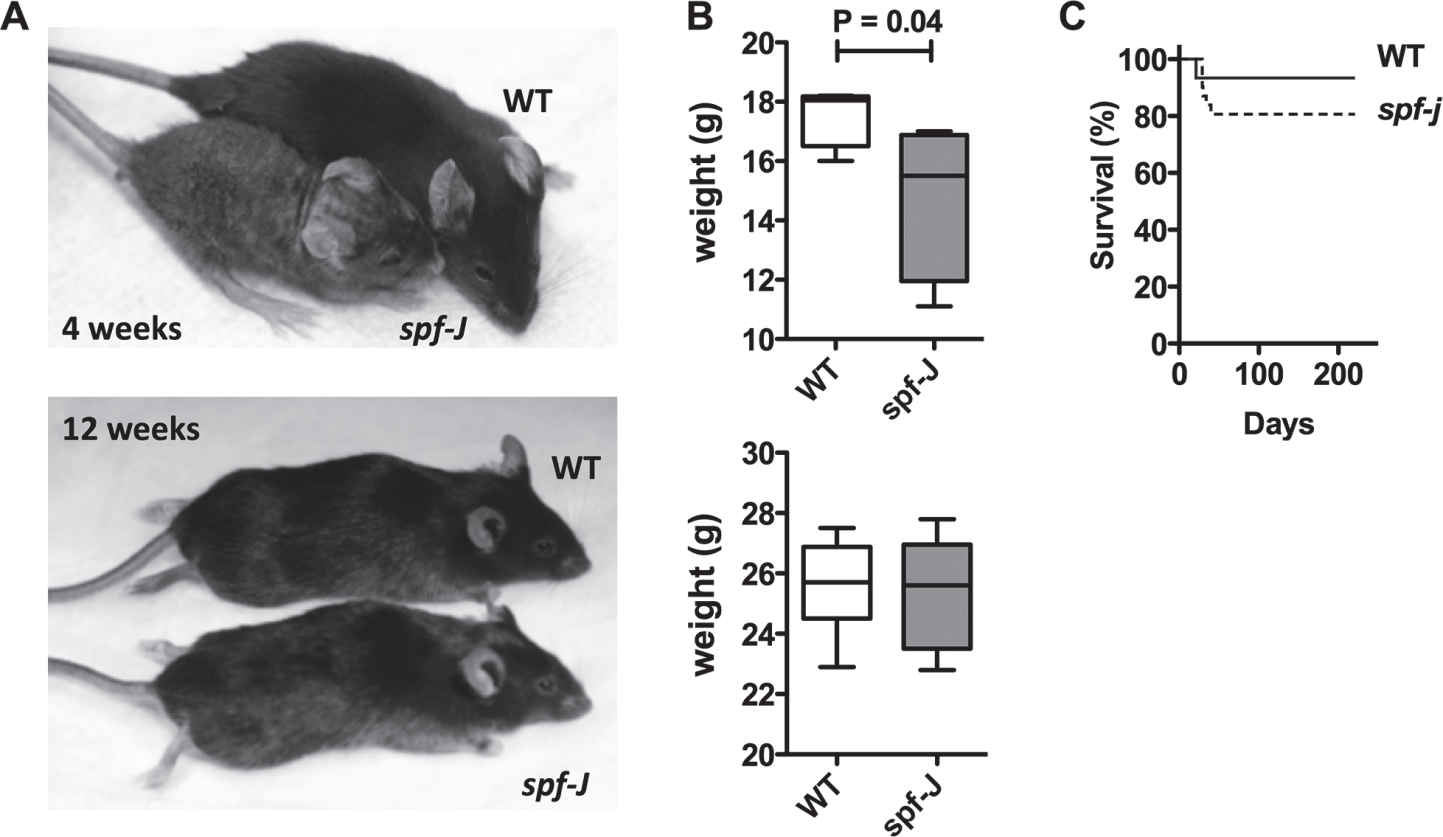
Phenotypic appearance, weight and survival on *spf-J* males. A) Photos of *spf-J* at 4 weeks (top) and 12 weeks (bottom). B) Weight of *spf-J* (N = 8) and WT (N = 9) at 4 weeks (top) and 12 weeks (bottom). C) Morbidity analysis for *spf-J* (N = 10) versus WT littermates (N = 10). Horizontal hatched bar indicates P < 0.05. WT – wild-type.

We were able to identify *spf-J* animals by coat and skin abnormalities (Fig. 2A) based on phenotype: *spf-J* appeared smaller than WT littermates (P = 0.04) and their coat was patchy with grey fur at 4 weeks of age. Despite these early differences, by 12 weeks of age, the size discrepancy disappeared and the coat color and density became similar to WT, making visual identification difficult (Figs. 2A and 2B). Consistent with the improvement in coat phenotype and weight, suggesting a milder phenotype, *spf-J* also showed comparable survival when compared to WT (Fig. 2C). The survival curves were not statistically distinguishable: *spf-J* showed an approximately 80% survival out to 220 days compared to 93% for unaffected WT (P = 0.20). This is in stark comparison to the *spf-ash* model of OTCD, which has about 15% survival at about 200 days [20]. In addition to increased survival, *spf-J* also display fecundity. Each hemizygous male is capable of producing litters that are comparable for the B6 background (data not shown). Overall, these data suggest that although *spf-J* shares some early visible phenotypic characteristics with *spf-ash, spf-J* mice show improved survival and a recovery of linear growth, weigh and coat texture.

### Reduced OTC protein and enzyme activity

**Figure 3:**
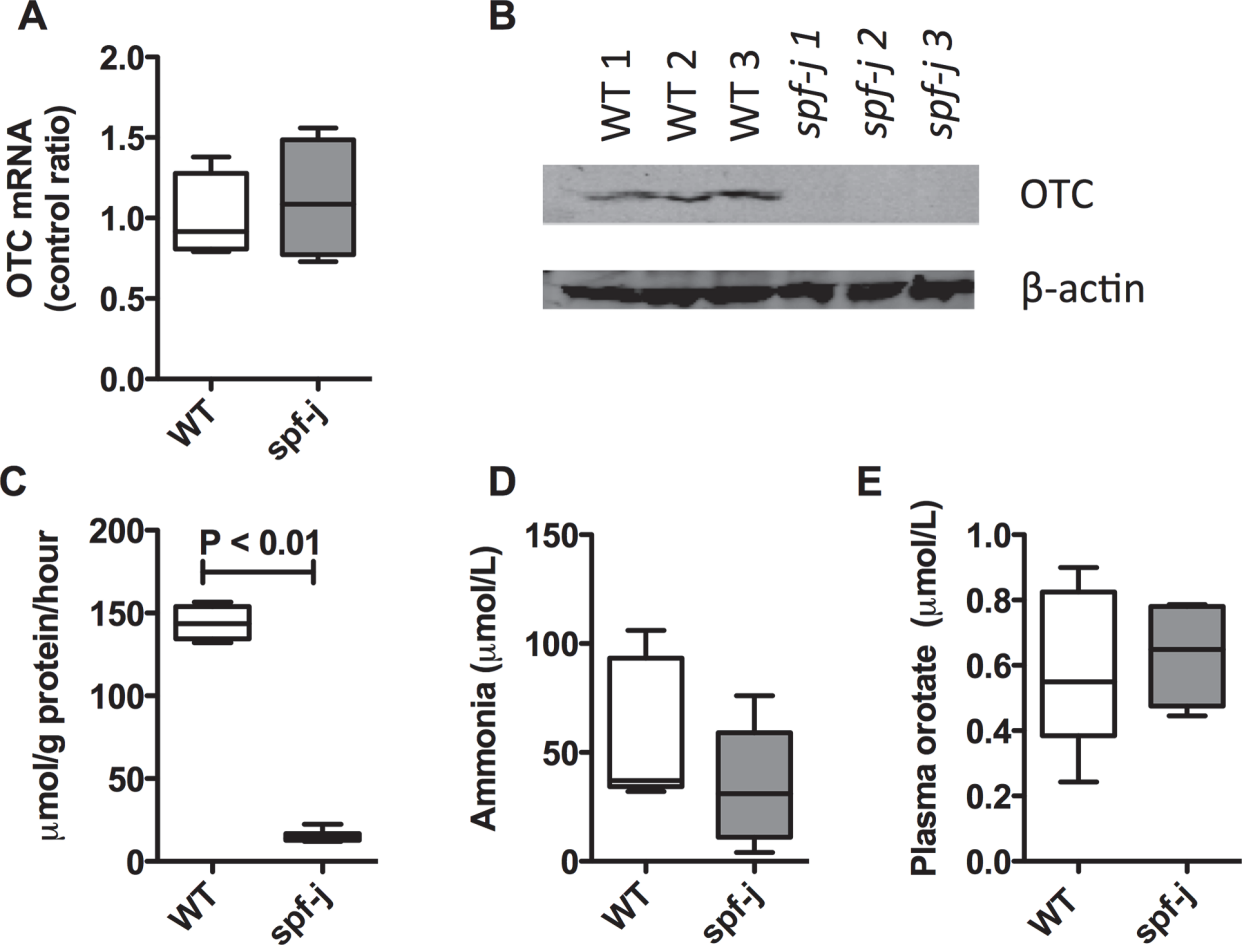
OTC enzyme and biochemical parameters in *spf-J* males. *Spf-J* and WT were maintained on a regular chow diet (22% protein) and euthanized at 8 weeks. Blood was collected by retro-orbital bleeding with plasma separated and stored at −80°C until use. Tissues were snap frozen and stored at −80°C until use. A) mRNA level in *spf-J* (N = 4) and WT (N = 4) livers. B) Immunoblot of OTC protein. C) OTC enzyme activity in liver homogenates from *spf-j* (N = 6) and WT (N = 4). D) Plasma ammonia in *spf-j* (N = 7) and WT (N = 6). E) Plasma orotate in *spf-j* (N = 5) and WT (N = 6). Hatched bar indicates P < 0.05. WT – wild-type.

Given the longer survival, we hypothesized that *spf-J* would display milder enzymatic findings. To characterize *Otc* K80N, we examined hepatic OTC mRNA, protein and enzyme activity (Fig. 3A–C). Livers from 8-week-old *spf-J* were harvested after euthanasia, and snap frozen. Similar levels of OTC mRNA (Fig. 3A) were seen by qRT-PCR in *spf-J* and WT. In contrast, the amount of OTC protein in *spf-J* was drastically reduced to below the detectable limit by immunoblot (Fig. 3B). Due to this drastic reduction in hepatic OTC protein, we next measured enzyme activity in liver homogenates. Consistent with reduced amounts of OTC protein by immunoblot, OTC enzyme activity was 10–12% of WT (P < 0.01). This enzyme activity level is similar to the previously reported case of late-onset OTCD mentioned above [19]. Overall, our protein and enzyme findings in the *spf-J* are consistent with the milder phenotype we observed.

### Biochemical features of *spf-J* at baseline

*Spf-ash*, another model of OTCD, display features of hyperammonemia and orotic aciduria at baseline [21]. Since the observable phenotype and reduction in enzyme activity was milder in *spf-J*, we hypothesized that the biochemical hallmarks of OTCD would be milder or absent at baseline. *Spf-J* and WT were maintained on normal mouse chow (22% protein). Blood was collected and analyzed for plasma ammonia, plasma orotate and plasma amino acids (Fig. 3D and 3E, Table 1). Liver and cerebrum were harvested and analyzed for tissue amino acids (Tables 2–3). On normal chow, *spf-J* failed to show differences in plasma ammonia or plasma orotate (Figs. 3D and 3E). When plasma amino acids were measured, some significant differences were seen (Table 1). In *spf-J*, glutamine, a metabolic sink for ammonia [22], was mildly increased over WT (768.3 μM versus 654.7 μM, P <0.01). Urea cycle intermediates ornithine (73.4 μM versus 95.3 μM, P < 0.01), citrulline (39.7 μM versus 95.4 μM, P < 0.01) and arginine (96.2 μM versus 138.4 μM, P <0.01) were significantly lower in *spf-J* versus WT, consistent with a proximal urea cycle defect. Branched chain amino acids valine, leucine and isoleucine were also significantly decreased (P≤0.02). In the *spf-j* liver (Table 2), only a few amino acid perturbations were seen: decreased aspartate (63.2 μmol/100g versus 94.5 μmol/100g) and glutamic acid (52.6 μmol/100g versus 72.8 μmol/100g), and mildly decreased ornithine (30.0 μmol/100g versus 36.3 μmol/100g). Interestingly, in the cerebrum (Table 3), 15 of the 20 amino acids measured were increased in *spf-j* including two well-established neuroactive amino acids, glycine (148.2 μmol/100g versus 119.9 μmol/100g, P = 0.02) and glutamine (350.3 μmol/100g versus 235.0 μmol/100g, P = 0.04), suggesting altered cerebral amino acid metabolism or transport at baseline in *spf-j*.

**Table 1:** Plasma amino acid concentrations at baseline.

	**WT**			**Spf-j**			
	**μM**	**SD**	**N**	**μM**	**SD**	**N**	**P-value**
**Taurine**	475.1	74.8	9	435.9	37.2	10	0.15
**Aspartic Acid**	20.1	3.2	9	19.3	3.1	10	0.58
**Hydroxyproline**	17.4	8.5	9	18.0	15.3	10	0.92
**Threonine**	219.6	19.7	9	185.4	13.5	10	<0.01
**Serine**	152.8	15.7	9	147.7	14.1	10	0.46
**Asparagine**	45.1	8.7	9	39.6	14.0	10	0.32
**Glutamic Acid**	40.2	12.3	9	36.9	7.7	10	0.48
**Glutamine**	654.7	67.1	9	768.3	28.2	10	<0.01
**Proline**	115.7	17.4	9	105.4	10.6	10	0.13
**Glycine**	302.1	63.7	9	253.2	55.3	10	0.09
**Alanine**	458.1	48.8	9	463.1	52.7	10	0.83
**Citrulline**	95.4	18.6	9	39.7	20.2	10	<0.01
**Valine**	251.6	19.5	9	216.6	19.1	10	<0.01
**Methionine**	87.7	7.9	9	78.9	11.7	10	0.08
**Isoleucine**	99.8	12.2	9	75.3	8.2	10	<0.01
**Leucine**	153.6	35.5	9	122.6	15.6	10	0.02
**Tyrosine**	116.2	12.5	9	106.8	13.7	10	0.14
**Phenylalanine**	74.4	6.7	9	68.8	8.7	10	0.14
**Homocystine**	0.6	0.9	9	0.1	0.3	10	0.14
**Ornithine**	95.3	14.0	9	73.4	8.3	10	<0.01
**Lysine**	386.4	39.5	9	331.1	41.0	10	<0.01
**Histidine**	75.4	8.0	9	79.7	8.8	10	0.29
**Arginine**	138.4	19.8	9	96.2	26.3	10	<0.01

Plasma was collected in 8-week-old *spf-j* and WT and snap frozen until analysis. Amino acid concentrations were determined by an amino acid analyzer and expressed as μmol/L. T-test with P < 0.05.. WT – wild type, SD – standard deviation.

**Table 2:** Liver amino acid concentrations at baseline.

	**WT**			**Spf-j**			
	**μmol/100 g**	**SD**	**N**	**μmol/100 g**	**SD**	**N**	**P-value**
**Taurine**	82.5	9.4	5	77.2	14.3	5	0.51
**Aspartic Acid**	94.5	11.0	5	63.2	12.4	5	<0.01
**Threonine**	41.5	1.8	5	34.3	7.5	5	0.07
**Serine**	59.0	3.3	5	49.2	10.3	5	0.07
**Asparagine**	26.8	2.9	5	22.2	3.7	5	0.06
**Glutamic Acid**	72.8	14.4	5	52.6	9.2	5	0.03
**Glutamine**	74.3	5.8	5	77.3	11.0	5	0.61
**Proline**	33.0	2.1	5	27.4	6.3	5	0.10
**Glycine**	84.2	5.8	5	74.0	9.9	5	0.08
**Alanine**	114.8	12.0	5	104.4	17.0	5	0.30
**Valine**	43.4	2.0	5	36.3	7.2	5	0.07
**Methionine**	18.8	1.2	5	15.6	3.1	5	0.07
**Isoleucine**	23.2	1.2	5	19.3	4.1	5	0.08
**Leucine**	44.3	2.4	5	38.0	6.4	5	0.07
**Tyrosine**	17.4	2.7	5	14.0	4.1	5	0.16
**Phenylalanine**	21.3	1.2	5	17.8	3.4	5	0.06
**Ornithine**	36.3	1.6	5	30.0	5.2	5	0.03
**Lysine**	51.2	2.6	5	45.2	7.0	5	0.11
**Histidine**	25.1	1.3	5	21.5	3.4	5	0.06
**Arginine**	0.8	0.3	5	0.6	0.3	5	0.25

Liver tissue was collected in 8-week-old *spf-j* and WT and snap frozen until analysis. Amino acids concentrations were determined by an amino acid analyzer and expressed as μmol/100 g tissue. T-test with P < 0.05. WT – wild type, SD – standard deviation.

**Table 3:** Cerebral amino acid concentrations at baseline.

	**WT**			**Spf-j**			
	**μmol/100 g**	**SD**	**N**	**μmol/100 g**	**SD**	**N**	**P-value**
**Taurine**	215.0	9.9	5	238.4	35.9	5	0.17
**Aspartic Acid**	245.2	31.9	5	294.7	52.0	5	0.11
**Threonine**	28.9	2.0	5	33.8	3.2	5	0.02
**Serine**	67.0	3.4	5	82.2	9.7	5	0.01
**Asparagine**	10.0	1.0	5	12.7	1.1	5	<0.01
**Glutamic Acid**	218.9	8.6	5	244.5	41.7	5	0.21
**Glutamine**	235.0	18.7	5	350.3	105.1	5	0.04
**Proline**	17.0	0.8	5	21.3	2.3	5	<0.01
**Glycine**	119.9	3.1	5	148.2	22.3	5	0.02
**Alanine**	87.2	12.2	5	104.6	24.8	5	0.19
**Valine**	13.4	1.0	5	16.5	1.9	5	0.01
**Methionine**	7.4	0.3	5	8.6	1.0	5	0.03
**Isoleucine**	8.5	0.6	5	10.2	1.2	5	0.02
**Leucine**	18.6	1.0	5	21.9	2.8	5	0.03
**Tyrosine**	8.7	0.6	5	12.0	2.6	5	0.03
**Phenylalanine**	9.7	0.3	5	11.8	1.8	5	0.03
**Ornithine**	1.0	0.2	5	1.3	0.2	5	0.03
**Lysine**	23.2	1.3	5	28.4	2.8	5	<0.01
**Histidine**	9.8	0.3	5	12.6	2.2	5	0.02
**Arginine**	23.4	0.7	5	24.8	3.6	5	0.41

Cerebral tissue was collected in 8-week-old *spf-j* and WT and snap frozen until analysis. Amino acid concentrations were determined by an amino acid analyzer and expressed as μmol/100 g tissue. T-test analysis with P < 0.05. WT – wild type, SD – standard deviation.

### *Spf-J* may be provoked to accumulate orotate

**Figure 4:**
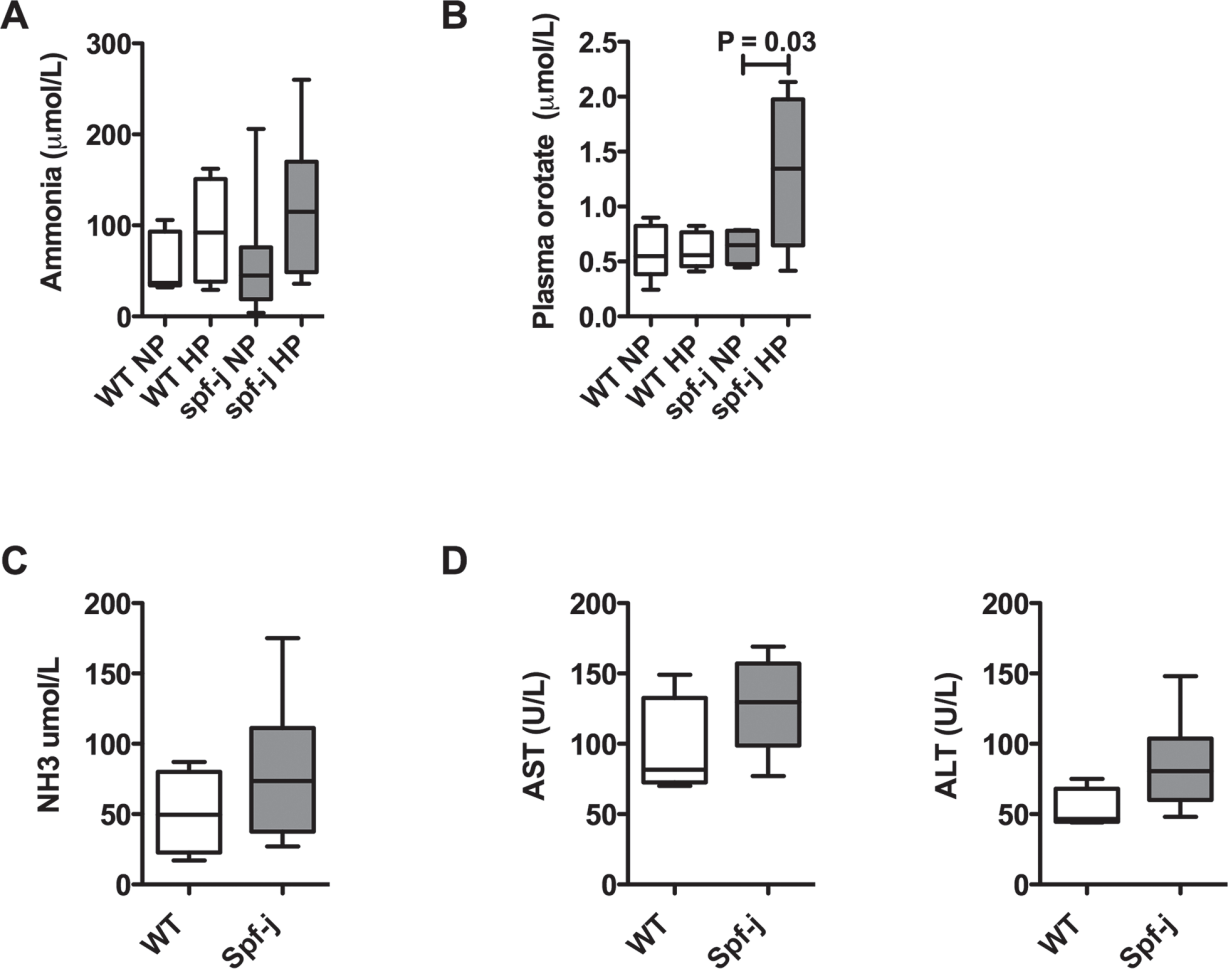
Urea cycle perturbations in *Spf-J* males. *Spf-J* (8 weeks) were maintained on a high protein chow diet (70% protein) and euthanized after 2 weeks (N = 6 / group). Blood was collected by retro-orbital bleeding with plasma separated and stored at −80°C until use. WT – wild-type, NP – normal protein, HP – high protein. A) Plasma ammonia and B) Plasma orotate for *spf-j* and WT. For immune challenge, 8-week-old *spf-J* (N = 6) and WT (N = 4) received an intraperitoneal injection of poly I:C (100 μg) once a day for 3 days. Tissues harvested and snap frozen and stored at −80°C until use. C) Plasma ammonia. D) Liver transaminases. Hatched bar indicates P < 0.05. WT – wild-type.

With the plasma amino acid profile suggesting perturbations in the biochemical phenotype at baseline, we next tried to provoke hyperammonemia and orotic aciduria. *Spf-J* and WT littermates were maintained on a high protein diet for 2 weeks (Figs. 4A and 4B). At the end of 2 weeks the animals were bled retro-orbitally and plasma ammonia and orotate were determined. Although we were not able to increase plasma ammonia significantly in *spf-J*, plasma orotate increased 2.5 – 3X over baseline in *spf-J* (P = 0.03). Therefore, *spf-J*, while displaying mild plasma biochemical abnormalities at baseline, may be provoked by protein challenge to display one of the biochemical hallmarks of OTCD, orotate accumulation.

### Amino acid perturbations following polyinosinic:polycytidylic acid (poly I:C )challenge

**Figure 5:**
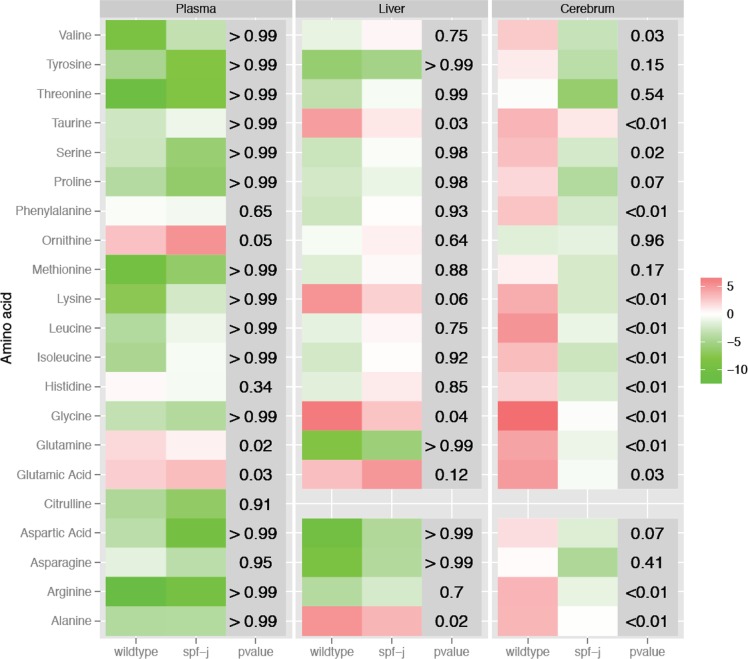
Changes in amino acids from baseline during immune challenge. Spf-J (N = 5) and WT (N = 5) received an intraperitoneal injection of poly I:C (100 μg) once a day for 3 days. Tissues harvested and snap frozen and stored at −80°C until use. Heat maps were constructed as described in **Methods** to represent the change in amino acid concentrations from baseline for plasma (left panel), liver (middle panel) and cerebrum (right panel). P-value < 0.05 was set as the threshold for significance.

Patients with urea cycle disorders may experience episodes of metabolic instability termed acute metabolic decompensation. To simulate viral infection with a systemic inflammatory response in *spf-j* mice, intraperitoneal poly I:C was given once a day for 3 days [23]. Following injection with poly I:C, *spf-j* mice fail to show differences in plasma ammonia (Fig. 4C) and liver transaminases (Fig. 4D). To explore amino acid perturbations, plasma, liver and cerebral amino acids were measured. Heat maps were constructed showing the change from baseline for each tissue (Fig. 5) with P-values reflecting the significance in differences of change. In plasma, there was a similar general decrease in amino acids from baseline in both *spf-j* and WT (Fig. 5, left panel, P > 0.05). Exceptions included glutamine, which displayed a greater increase over baseline in WT (P = 0.03), and glutamic acid, which displayed a greater increase over baseline in *spf-j* (P = 0.03). Similar to plasma, most of the changes from baseline in liver were not statistically different (Fig. 5, center panel, P > 0.05). Alanine (P = 0.02), glycine (P = 0.04) and taurine (P = 0.03) were the exceptions, having greater increases from baseline in WT. Unlike plasma and liver, the effect of poly I:C treatment in the brain was more pronounced (Fig. 5, right panel). While WT showed an increase from baseline in cerebral amino acids following poly I:C injection (red), *spf-j* trended in the opposite direction for many amino acids including valine, serine, phenylalanine, lysine, leucine, isoleucine, histidine, glutamine, glutamic acid and arginine ( green, P < 0.05). In addition, taurine, glycine and alanine remained relatively static in *spf-j* (P < 0.01) while increasing in WT.

## Discussion

Due to the inheritance of OTCD, the phenotypic expression of the disease severity in patients is dependent upon the nature of the mutation, genetic background and in females, X-inactivation in the liver. In males, this disorder classically manifests with symptomatic hyperammonemia in infancy, although milder alleles have been described [12,13,19]. In addition, phenotypic variability can be seen in males within the same family with the same mutation suggesting modifier alleles may play a role [24].

Late-onset symptoms of OTCD, due to milder enzyme defects, may manifest in men as late as the fifth or sixth decade [25] given the right precipitant, such as the Atkins’ diet [26]. Galloway et al. described a previously healthy male who experienced his first documented hyperammonemic episode at 13 years of age [19]. The precipitant of this episode was unknown. On admission to hospital, his ammonia was 750 μmol/L and hemodialysis was initiated. Urine orotate was 813 μmol/mmol creatinine, suggesting a diagnosis of OTCD. A subsequent liver biopsy demonstrated OTC enzyme activity to be 11% of normal. Molecular investigation revealed a lysine substitution for asparagine (K80N) in the OTC protein. This amino acid substitution in the OTC enzyme is identical to the mutation described here in the *spf-J* mouse (Fig. 1A). Similar to the reported patient, the K80N mutation results in a reduction in enzyme activity to ∼11–12% in *spf-J* (Fig. 3C). The mutation lies outside the substrate binding and catalytic regions of the enzyme [13] and may be related to homo-oligomerization or stability of the enzyme. In our studies, we demonstrated normal levels of OTC mRNA (Fig. 3A) and OTC protein that was below the limit of detection by immunoblot (Fig. 3B). These findings lead us to suggest that the K80N mutation may destabilize the enzyme, leading to its early degradation.

In general, mouse models of UCD have yielded insights into disease pathophysiology, the influence of genetic background and the evaluation of novel therapeutics. Two animal models of OTCD, the *sparse-fur (spf)* and the *sparse-fur abnormal skin and hair (spf-ash)*, have been used to study disease pathogenesis and to evaluate therapies (reviewed in [21]). The biochemical characteristics of both of these models include OTCD, elevated plasma ammonia and glutamine, low plasma citrulline and arginine, and elevated urinary orotic acid excretion. In contrast to these previously described models, the *spf-J* displays a milder phenotype: normal ammonia and plasma orotate at baseline (Figs. 3D and 3E), a small elevation in plasma glutamine, and mild depressions of plasma ornithine, citrulline and arginine (Table 1). The elevated plasma glutamine seen in *spf-J* at baseline (Table 1) suggests that ammonia disposal is indeed compromised and that glutamine synthetase may provide an alternative route of elimination. Consistent with this assertion is the depletion of plasma branched chain amino acids, which may be consumed to support glutamine synthesis (reviewed in [27]).

Patients with urea cycle disorders experience episodes of acute metabolic decompensation characterized by hyperammonemia. Viral infection is the most common cause of acute metabolic decompensation and is associated with markers of increased morbidity [28]. The viral infection mimic, poly I:C was not sufficient to produce hyperammonemia consistently in *spf-j* (Fig. 4C). However, tissue amino acid perturbations could be seen. Although both WT and spf-j displayed similar trends in plasma amino acids (Fig. 5, left and center panels), cerebral amino acids were more discrepant between animals during poly I:C treatment (Fig. 5, right panel): WT increased, while *spf-j* decreased cerebral amino acids. These findings, combined with the elevation of cerebral amino acids at baseline (Table 3), supports the hypothesis of altered cerebral amino acid metabolism or transport in *spf-j*. In the brain, amino acids and their derivatives play a role in synaptic transmission by serving as a source of energy, precursors to neuro-active compounds, allosteric regulators, and even neurotransmitters [29,30]. Several amino acids involved in various aspects of neurotransmission failed to increase or were depressed in *spf-j* including glycine (excitatory and inhibitory neurotransmission), histidine (source of histamine) and arginine (source of nitric oxide). These perturbations in the cerebral amino acid pool may be due to decreased transport across the blood brain barrier, depressed synthesis, or increased metabolism [31]. Being that many amino acids are precursors or cofactors for neurotransmission, one could postulate that these perturbations in cerebral amino acids may lead to neurocognitive deficits. Indeed, alterations in cognitive functioning seen in asymptomatic OTC carriers suggest that other factors besides hyperammonemia may play a role [32]. This avenue of inquiry should be explored and necessitates defining the neurocognitive phenotype of *spf-j* and the relationship between systemic inflammation and brain amino acid metabolism.

In conclusion, the *spf–J* has the benefits of a pure inbred strain, allowing for the ideal characterization of this mild OTC mutation on a uniform genetic background. Added benefits include fecundity and long-term survival. Due to the robust nature of these animals, this model is ideal for studying interactions with other metabolic pathways and mapping of genes that modulate disease activity. Regarding therapeutic approaches, the instability of the enzyme suggests that this model may serve as a target for the preclinical evaluation of small molecule chaperones or activators. From a pathophysiologic standpoint, we anticipate that this model may also be useful for studying cerebral amino acid metabolism and transport and the long-term effects of mild OTCD on neurodevelopment and IQ. Among the many human mutations that lead to a pathological condition, few have a spontaneous orthologous equivalent in the mouse and vice versa. In this regard, the *spf-J* mouse is a unique tool for studying mild OTC deficiency.
